# Prevalence of Livestock-Associated MRSA in Communities with High Pig-Densities in The Netherlands

**DOI:** 10.1371/journal.pone.0009385

**Published:** 2010-02-25

**Authors:** Brigitte A. van Cleef, Erwin J. M. Verkade, Mireille W. Wulf, Anton G. Buiting, Andreas Voss, Xander W. Huijsdens, Wilfrid van Pelt, Mick N. Mulders, Jan A. Kluytmans

**Affiliations:** 1 Centre for Infectious Disease Control Netherlands, RIVM National Institute for Public Health and the Environment, Bilthoven, The Netherlands; 2 Department of Medical Microbiology, VU University Medical Centre, Amsterdam, The Netherlands; 3 EU-HEALTH Project PILGRIM of the 7th Framework Programme, Nijmegen, The Netherlands; 4 Laboratory for Microbiology and Infection Control, Amphia Hospital, Breda, The Netherlands; 5 PAMM Laboratory of Medical Microbiology and Catharina Hospital Eindhoven, Veldhoven, The Netherlands; 6 Laboratory for Medical Microbiology and Immunology, St. Elisabeth Hospital, Tilburg, The Netherlands; 7 Department of Medical Microbiology and Infection Control, Canisius-Wilhelmina Hospital, Nijmegen, The Netherlands; 8 Department of Medical Microbiology, Radboud University Nijmegen Medical Centre, Nijmegen, The Netherlands; National Institutes of Health, United States of America

## Abstract

**Background:**

Recently, livestock-associated methicillin-resistant *Staphylococcus aureus* CC398 has been discovered in animals, livestock farmers and retail meat. This cross-sectional study aimed to determine the spread to persons not in direct contact with livestock in areas with a high density of pig farms.

**Methodology/Principal Findings:**

With a random mailing in 3 selected municipalities in the Netherlands, adult persons were asked to fill in a questionnaire and to take a nose swab. In total, complete information was obtained on 583 persons. Of the 534 persons without livestock-contact, one was positive for MRSA (0.2%; 95% confidence interval, <0.01–1.2). Of the 49 persons who did indicate to be working at or living on a livestock farm, 13 were positive for MRSA (26.5%; 95% confidence interval, 16.1–40.4). All *spa*-types belonged to CC398.

**Conclusions/Significance:**

Livestock-associated MRSA has a high prevalence in people with direct contact with animals. At this moment it has not spread from the farms into the community.

## Introduction

Traditionally, methicillin-resistant *Staphylococcus aureus* (MRSA) has been considered a hospital-associated pathogen. Recently, the epidemiology of MRSA has changed from the confined settings of the hospital to the general population. Community-associated MRSA has been shown to cause severe infections in previously healthy persons [1].

A new development is the emergence of a distinct clone of MRSA that is related to an extensive reservoir in pigs and cattle. It was first recognized in the Netherlands in 2003 [2]. As this clone was found to be non-typable by pulsed-field gel electrophoresis (PFGE) with *Sma*I, it was originally called NT-MRSA [3]. Further research revealed that all of these strains belonged to multilocus sequence type clonal complex (CC) 398 [4]. A subsequent case-control study confirmed that people in contact with pigs and veal calves were more prone to carry MRSA CC398 [5]. At present it is clear that people who have frequent contact with live pigs and veal calves have extremely high carriage rates (prevalence 25–35%) [6]. By the end of 2008, 42% of all newly detected MRSA strains in the Netherlands were CC398, up from 30% by the end of 2007 (www.rivm.nl/mrsa).

A recent survey by the Food and Consumer Product Safety Authority in the Netherlands (VWA) found MRSA on 11% of the meat samples in retail (with a minimum MRSA prevalence of 3% in game and a maximum of 31% in turkey) [7]. Other studies confirmed the contamination of meat with MRSA, although the prevalence varied (2.5% [8], 17% [9], 0.7% [10], 5% [11], 0% [12] and 17% R. de Jonge, J.E. Verdier and A.H. Havelaar, submitted). So far, a relation between eating meat and MRSA-carriage is not found, but it is of concern that this type of MRSA has entered in the food chain and handling of meat could thus become a mode of acquisition of MRSA.

Meanwhile, serious invasive infections from Europe, Asia and America due to MRSA CC398 have been reported [5], [13]–[18]. In hospitals in husbandry-dense areas in the Netherlands, the majority of newly identified MRSA carriers are CC398 [19], and the first outbreak with MRSA CC398 in hospitals has been reported [20]. This means that MRSA is not only a human pathogen, but also a zoonotic pathogen, particularly affecting people working in animal husbandry.

In order to get an idea of the magnitude of the problem, knowledge on the exact spread of this specific clone in the general community is desired. The current study aimed and succeeded to determine if MRSA CC398 has spread from the farms into the rest of the community in areas with an extremely high density of pig farms.

## Materials and Methods

### Ethics Statement

The medical ethical committee of the St. Elisabeth Hospital in Tilburg approved the study.

### Enrollment

**Figure 1:**
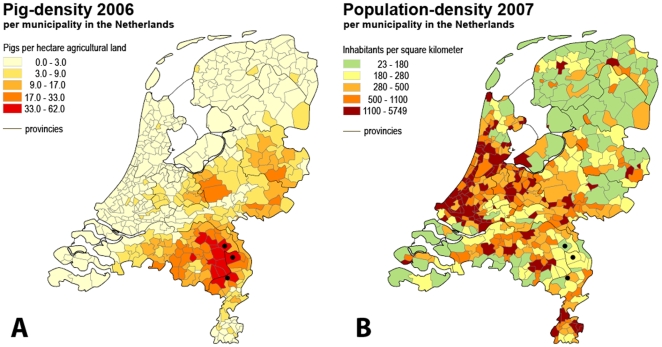
The pig-density and population-density in the Netherlands. Pig-density is depicted in panel A, population-density is depicted in panel B. The participating municipals of St. Anthonis, Venray and Meijel are indicated with “•”. Source: CBS Statistics Netherlands (www.cbs.nl).

This cross-sectional study was conducted between July 2008 and January 2009 in three municipalities from the area with the highest density of pigs in the Netherlands, i.e. Venray, St. Anthonis and Meijel. They are located in the southeast of the Netherlands with a relatively low human population-density and a pig-density of approximately 3,000 pigs per square kilometer [21] (Figure 1). A random sample of adult persons (≥18 years of age) from the local registry of inhabitants was taken. The sample was stratified for age and gender according to the characteristics of the general population of the Netherlands. Stratification to livestock-contact was not performed in order to prevent response bias.

### Sample Size

The sample size was calculated, based on the following assumptions. The background prevalence of MRSA was assumed to be less than 0.5% [22]–[24]. To confirm that the prevalence of MRSA in persons living in pig-dense areas without livestock-contact is 2% or more with an alpha-error of 0.05 and a beta-error of 0.10, the estimated sample size was 450 persons who had no contact with livestock. After correction for livestock-contact (25%) and non-response (75%), a questionnaire was mailed to 2703 people. The following questions had to be answered: age, gender, living at a livestock farm, contact with livestock, working in healthcare, past history of MRSA, contact with known MRSA positive persons in the last year and hospitalization abroad in the last six months (Figure S1). Participants were asked to supply a written informed consent.

### Samples and Microbiological Procedures

Subsequently, appropriate transport medium and instructions for sampling were supplied by mail to the participants. A nasal swab was taken by the subjects themselves and sent by mail to one of the participating microbiology laboratories to determine the presence of MRSA. Nasal swabs were inoculated on Columbia blood agar plates with 5% sheep blood to check for adequate sampling and subsequently enriched in Mueller-Hinton broth containing 6.5% NaCl. Both media were incubated for 24 h at 35°C. From the overnight Mueller-Hinton broth, 10 µl was streaked onto MRSA ID (bioMérieux, La Balme Les Grottes, France) agar plates with a sterile loop using a three-streak dilution method. The results were read after 20 h of incubation at 35°C. Growth of colonies showing green coloration was considered to be indicative for MRSA. Colonies with colors other than green, or no growth at all were considered negative. The procedure was performed as recommended by the manufacturer. Green colonies were confirmed to be MRSA by latex agglutination [25], cefoxitin disk diffusion [26] and duplex PCR (*mec*A gene and the *S. aureus* specific target Martineau-sequence). In addition, staphylococcal protein A (*spa*) typing was conducted according to Harmsen et al. [27]. Resistance profiles to 21 antimicrobial agents of all confirmed MRSA strains were determined with the VITEK system (bioMérieux SA, Craponne, France) according to the manufacturer's instructions.

### Statistical Analyses

MRSA prevalence rates with Wilson's 95% confidence intervals (CI) were reported separately for persons with and without livestock-contact, based on information from the questionnaire. Contacted persons were compared to responders with Wilson signed rank and chi-square tests for age and gender categories. Possible determinants for MRSA carriage – apart from livestock-contact – were calculated with crude univariate and adjusted multivariate odds ratios with logistic regression.

## Results

**Figure 2:**
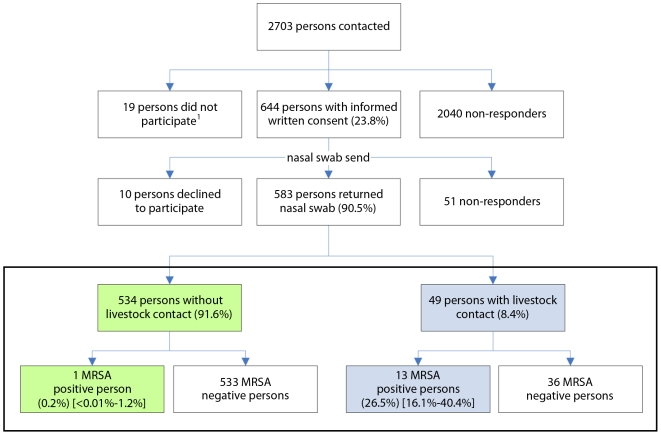
Flow chart of the study procedure and major results. Major study results are depicted in the box. ^1^Nineteen persons with incomplete response: 9 persons returned the questionnaire but not the informed written consent, 5 persons declined to participate, 2 persons died and 3 persons returned the informed consent after the deadline.

The flow chart of the study procedure is depicted in figure 2. Of the 2703 persons contacted for participation, 644 persons (23.8%) returned their informed consent form and questionnaire. From these persons, 583 (90.5%) returned the nasal swab to the microbiological laboratory. All nasal swabs grew micro-organisms on the Columbia blood agar plates, indicative for adequate sampling.

The median age of the 583 participants was 50 years (interquartile range (IQR) 21 years, total range 18–91 years), significantly higher than that of the contacted persons (n = 2703, median 46 years, IQR 26 years, p<0.001). The percentage of men in the 583 participants was 42.7%, which is significantly (p = 0.006) lower compared with 49.0% in the contacted group. Specifically, men of 18–40 years of age enrolled to a lesser extend in the study (data not shown).

Of the 534 persons without livestock-contact only one person (0.2%; 95% CI<0.01–1.2) tested positive for MRSA (Figure 2). In contrast, thirteen (26.5%; 95% CI 16.1–40.4) of the 49 persons with livestock-contact (either work at or live on a livestock farm) tested positive for MRSA. Eleven of the 13 MRSA positive persons reported contact with pigs, one with veal calves and one with poultry. Four had been tested positive for MRSA previously, and 7 out of 13 had reported recent contact with MRSA positive persons. None of the other factors asked for in the questionnaire (working in healthcare, hospitalization abroad) was a significant risk factor for carriage of MRSA, in both the univariate and multivariate analysis.

All recovered MRSA strains have *spa*-types that belong to the known livestock-associated clone CC398 [28]. Antibiotic resistance patterns also grossly correspond with MRSA CC398, being uniformly resistant to tetracycline (Table 1).

**Table 1:** *Spa*-types and antibiotic resistance patterns of the recovered MRSA strains.

Contact with	*Spa*-type	te	tr	er	cl	ge	to	ci	ni	va	ri	fu	li	mu
veal calves	t011	R	R	S	S	R	I	S	S	S	S	S	S	S
pigs	t011	R	S	R	R	S	S	S	S	S	S	S	S	S
pigs	t011	R	R	R	R	R	I	S	S	S	S	S	S	S
pigs	t011	R	S	R	R	S	S	S	S	S	S	S	S	S
pigs	t011	R	R	R	R	I	I	S	S	S	S	S	S	S
pigs	t011	R	R	R	R	R	R	S	S	S	S	S	S	S
pigs	t011	R	S	S	S	S	S	S	S	S	S	S	S	S
pigs	t108	R	S	S	S	S	S	S	S	S	S	S	S	S
no livestock	t108	R	S	S	S	S	S	S	S	S	S	S	S	S
poultry	t108	R	R	R	R	R	I	R	S	S	S	S	S	S
pigs	t571	R	R	S	S	S	S	S	S	S	S	S	S	S
pigs	t2330	R	R	R	R	S	S	S	S	S	S	S	S	S
pigs	t2330	R	R	S	S	S	S	S	S	S	S	S	S	S
pigs	t2330	R	R	R	R	S	S	S	S	S	S	S	S	S

All *spa*-types belong to CC398 [28]. S = senstitive, R = resistant, I = intermediate sensitivity, te = tetracyclin, tr = trimethoprim/sulfamethoxazole, er = erythromycin, cl = clindamycin, ge = gentamicin, to = tobramycin, ci = ciprofloxacin, ni = nitrofurantoin, va = vancomycin, ri = rifampicin, fu = fusidic acid, li = linezolid, mu = mupirocin.

## Discussion

The 0.2% (95% CI<0.01–1.2) prevalence of carriage of MRSA among persons not reporting contact with livestock was low and comparable to that in the general population (<0.01–0.13%) [22]–[24]. The one *spa*-type found belonged to CC398, indicating an initial source in livestock. Since this person reported no direct contact with livestock, the route of transmission remains unclear. It could be indirect contact with a MRSA CC398 carrier or by possible environmental contamination. A recent study sampled 422 pupils from a secondary school in Germany not living on pig farms, and did not find any MRSA, which is comparable to this study [29].

Of the persons who reported contact with livestock, 26.5% were positive for MRSA. This is comparable to data found elsewhere, i.e. 26% and 14% in pig farmers and 12.5% in veterinarians attending an international pig health convention [2], [6], [30], but lower than found in a German study in pig farms (45%) and veterinarians (45%) [29]. This supports the present national guidelines in the Netherlands, which state that persons in regular contact with live pigs or veal calves should be screened for MRSA upon hospital admission. All MRSA strains in the present study had antibiotic susceptibility profiles comparable with other MRSA CC398 strains e.g. tetracycline-resistant and mupirocin-susceptible.

The main purpose of the present study was to investigate the potential spread of MRSA CC398 into the community. This can occur either through person to person spread or by contamination of the environment and it would be detected first in these areas with an extremely high pig-density. The current low prevalence in these communities is therefore reassuring.

Another potential route of transmission is through contaminated meat. MRSA has been found at a relatively high prevalence in retail meat samples (up to 17%). However, the amount of MRSA per sample was low (<10 colony forming units per gram meat) [7]. The risk that contaminated meat will cause spread of MRSA into the community is considered to be low [31]. In this study, we did not find any spread of livestock-associated MRSA in persons not having contact with livestock. Although we have no information on the dietary habits of the participants we assume that in a random sample most people will regularly eat meat. This indicates that the high prevalence of MRSA in retail meat does not contribute significantly to transmission of MRSA into the community at this time. Similar results were also found in other studies, that showed only high MRSA-carriage rates in persons in direct contact with livestock [5].

There are two limitations of this study. First, the chance for selection bias. The response on the first invitation letter was 23.8%, being grossly comparable to the response to other random mailing studies in the Netherlands (32%, 44% and 28% [32]–[34]). The response of persons invited to send a nasal swab was 90.5%, which is considered adequate. However, there were significant differences in gender and age between contacted persons and the subjects who participated. Earlier random mailing studies in the Netherlands dealing with unrelated topics reported the same deviations in response percentages; namely fewer men of 18–40 years of age [32]–[34]. Therefore, we consider the response in line with studies on unrelated topics and the chances for selection bias as negligible. In addition, this selection bias would only be of concern when one would expect that men of 18–40 years of age are at a higher risk for colonization with MRSA, compared to other gender and age groups. We currently have no reason to assume this.

Another possible limitation is nasal self-swabbing; since subjects have to swab their own nostrils, this may affect the quality of sampling. We checked for sampling adequacy by looking for the presence of micro-organisms in general. In addition, a recent study comparing samples taken by professional samplers and by individuals themselves showed excellent concordance of the results [35]. These results were confirmed in a short validation study performed by our own group (B. van Cleef, unpublished results). Therefore, the quality of the samples taken in the present study can be considered to be adequate. Nevertheless, checking for the carriage rate of *S. aureus* (approximately 30% in the general population) might have lessoned this limitation of nasal self-swabbing [36].

The outcome of this survey is reassuring, considering the potential impact of MRSA CC398 on public health, as there was very limited spread to persons without livestock-contact in areas with an extremely high pig-density. This lower transmissibility of MRSA CC398 compared to other MRSA strains was also found in hospital-based studies [19], [37]. These findings indicate that strains from CC398 are primarily adapted to animals and do not easily spread among humans. This would limit the impact of this recently emerged clone on public health.

In conclusion, MRSA CC398 has an extremely high prevalence in people who are in contact with livestock, but has not spread into the rest of the community at this time. Therefore, preventive measures should primarily be aimed at person who work with animals or live on farms.

## Supporting Information

Figure S1 Questionnaire used in this cross-sectional study.
(0.04 MB PDF)
