# Supplementary material for: Prevalence of Livestock-Associated MRSA in Communities with High Pig-Densities in The Netherlands
Source: PLoS One. 2010 Feb 25;5(2):e9385. doi: 10.1371/journal.pone.0009385 (PMC2828479; doi:10.1371/journal.pone.0009385)
Supplement: Figure S1 — Questionnaire used in this cross-sectional study. (0.04 MB PDF) [file pone.0009385.s001.pdf]

Supplementary Figure S1. Questionnaire used in this cross-sectional study

**General**		
**1**	Gender	❒ male ❒ female
**2**	Date of birth	……-……-………
**3**	Postal code	…………………..
**4**	Current date	……-……-………

**Living and working**		
**5a**	Are you living at a pig farm at the moment?	❒ yes ❒ no
**b**	Are you living at a veal calf farm at the moment?	❒ yes ❒ no
**c**	Are you living at a poultry farm at the moment?	❒ yes ❒ no
**6a**	Do you work with live pigs (at least once a week)?	❒ yes ❒ no
**b**	Do you work with live veal calves (at least once a week)?	❒ yes ❒ no
**c**	Do you work with live poultry (at least once a week)?	❒ yes ❒ no
**7**	Do you work in healthcare?	❒ yes ❒ no
	If so, in which function?	…………………………………………………………………

**Health**		
**8**	Have you ever had MRSA previously?	❒ yes ❒ no
	If so, are you MRSA negative at the moment?	❒ yes ❒ no
	If so, since when?	…………………………………………………………………
**9**	Have you had contact with a MRSA positive person *in the last year*?	❒ yes ❒ no
**10**	Were you admitted to a hospital abroad *in the last 6 months*?	❒ yes ❒ no

Comments: …………………………………………………………………………………..........
…………………………………………………………………………………..........

Thank you very much for completing the questionnaire!
